# Smarter AI, Healthier Students? How Perceived AI Assistant Intelligence Shapes Medical Students’ Mental Health Through Learning Goal Progress and Academic Anxiety

**DOI:** 10.3390/bs16101715

**Published:** 2026-09-22

**Authors:** Yue Chen, Shuyue Zhang, Xinru Liu, Liuyiran Li, Miao Si, Chenchen Xu

**Affiliations:** 1 The Fourth School of Clinical Medicine, Nanjing Medical University, Nanjing 211166, China; yfxj3201cy@126.com (Y.C.); qq2928413335@163.com (S.Z.); liuxinru@stu.njmu.edu.cn (X.L.); liliuyiran@163.com (L.L.); 2 Kangda College, Nanjing Medical University, Lianyungang 222000, China

**Keywords:** perceived AI assistant intelligence, learning goal progress, academic anxiety, mental health, AI literacy, medical students

## Abstract

As artificial intelligence (AI) adoption grows in medical education, AI assistants have become important learning support tools for medical students. However, existing research has primarily focused on learning performance and technology adoption, while little is known about how perceived AI assistant intelligence influences medical students’ mental health. Drawing on Self-Determination Theory (SDT), this study develops a model linking perceived AI assistant intelligence to mental health through the serial mediating roles of learning goal progress and academic anxiety, while examining the moderating role of AI literacy. A two-study design was employed. Study 1 surveyed 721 Chinese medical students, and Study 2 conducted a real human–AI interaction experiment with 398 medical students. Results showed that perceived AI assistant intelligence positively predicts learning goal progress. Learning goal progress and academic anxiety jointly mediate the relationship between perceived AI assistant intelligence and mental health. In addition, AI literacy strengthens the positive effect of perceived AI assistant intelligence on learning goal progress and enhances its indirect effect on mental health through the serial mediation pathway. The experimental findings further support the robustness of these relationships. This study extends research on AI in education by uncovering the psychological mechanisms through which perceived AI assistant intelligence affects medical students’ mental health. It also enriches the application of SDT in human–AI collaborative learning contexts and provides practical implications for optimizing AI-assisted learning environments, improving AI literacy, and promoting student well-being.

## 1. Introduction

Mental health problems are a significant concern in medical education and have long been a predominant issue (Quek et al., 2019). Compared with undergraduate students, medical students face the added burden of an increased course load, frequent evaluations, and complex clinical training (Rotenstein et al., 2016). Mental health problems such as anxiety, depression, stress, and burnout are common among medical students and can affect future healthcare services, as well as their academic performance and career progression (Dyrbye & Shanafelt, 2016; Quek et al., 2019). Thus, the factors that influence medical students’ mental health and how these can be addressed through interventions are a major research focus in medical education (Abd-Alrazaq et al., 2023). Meanwhile, artificial intelligence (AI) technology has been increasingly integrated into medical education, providing new forms of support for medical students in knowledge acquisition, exam preparation, clinical reasoning, and research skills development (Kasneci et al., 2023; Kung et al., 2023; Preiksaitis & Rose, 2023). However, not all AI assistants provide the same level of learning support, as they differ in their abilities to understand users’ needs and instructions, process and respond to information, and adapt their interactions to users’ specific needs (Bartneck et al., 2009; Shaw et al., 2025). In this study, perceived AI assistant intelligence refers to students’ overall appraisal of the intelligence demonstrated by an AI assistant, including its apparent ability to understand users’ needs and instructions, process and respond to information effectively, provide accurate and contextually appropriate responses, and adapt its interactions to support users’ tasks (Bartneck et al., 2009; Ling et al., 2025). In educational contexts, such intelligence may be reflected in the AI assistant’s ability to understand learning needs, provide personalized feedback, sustain meaningful interactions, and support learning-related decision-making. Thus, perceived AI assistant intelligence represents an important perceived characteristic of AI-assisted learning environments that may shape students’ learning experiences and outcomes.

AI assistants have been increasingly used in medical education, including knowledge acquisition, clinical reasoning training, and exam preparation, showcasing their potential to improve learning capabilities (Hale et al., 2024; Kung et al., 2023). Current studies, however, have mainly examined AI’s effects on outcome variables such as learning performance, technology adoption, and willingness to use, with little systematic understanding of how perceived AI assistant intelligence affects medical students’ mental health (Preiksaitis & Rose, 2023; Pupic et al., 2023). Existing studies indicate that AI systems can improve learning efficiency, enhance engagement, and provide personalized explanations, thereby improving learning outcomes (Tozsin et al., 2024; Zawacki-Richter et al., 2019). Thus, it is crucial to examine further the mechanisms through which perceived AI assistant intelligence influences medical students’ mental health.

Self-Determination Theory (SDT) holds that meeting three basic psychological needs—autonomy, competence, and relatedness—is important for people to continue investing in learning and to have a positive psychological experience (Deci & Ryan, 2000; Ryan & Deci, 2020). When the learning environment is supportive and effectively enables individuals to attain their learning objectives, students are likely to feel autonomous, competent, and supported, which affects positive learning behaviors and psychological adaptation (Baidoo-Anu & Ansah, 2023; Chen et al., 2023). In AI learning contexts, highly intelligent AI assistants can not only provide more precise and timely feedback but also offer recommendations based on learning needs, support learning decisions, and interact continuously (Kasneci et al., 2023; Preiksaitis & Rose, 2023). Students are likely to see these AI assistants as helpful learning tools that satisfy their needs for autonomy, competence, and relatedness. Previous studies suggest that an environment that fosters basic psychological needs can improve student learning engagement, self-regulated learning, and autonomous motivation, and can support positive learning outcomes (Bureau et al., 2022; Deci & Ryan, 2000; Howard et al., 2021).

This impact can initially show up in progress toward study goals. Study goal progress is an individual’s subjective assessment of how much they have achieved toward set study goals and how those goals have developed (Wanberg et al., 2010). In medical education contexts, highly intelligent AI assistants can provide medical students with personalized feedback, learning-planning assistance—such as setting learning goals, organizing learning tasks, and developing personalized study plans—and support for solving learning problems, thereby helping students make continuous progress toward their learning goals (Harkin et al., 2016). Study goal progress represents, beyond completing learning tasks, an increased sense of control and confidence in the learning process and is therefore important for medical students with high workloads and evaluation requirements in the long run (Klug & Maier, 2015). In addition, students’ emotional experiences are closely related to the progress toward study goals. Studies have shown that a sense of continuous improvement toward learning targets significantly influences well-being and perceived control, and reduces stress and anxiety by framing learning challenges as controllable, manageable tasks (Klug & Maier, 2015). Academic anxiety is a common negative academic emotion that not only negatively affects learning engagement and performance but also negatively affects mental health (Pekrun, 2006). Thus, highly intelligent AI assistants could further improve medical students’ mental well-being by helping them achieve their study objectives and minimizing academic stress, anxiety, and related issues.

However, not all students will benefit equally from highly intelligent AI assistants. AI literacy is a crucial factor in how effectively individuals use AI tools (Lintner, 2024; Long & Magerko, 2020; Ng et al., 2021). In general, students with higher AI literacy are more likely to interpret the AI output accurately, use it to develop learning materials more effectively, and feel greater autonomy, competence, and belongingness when using it (L. Wang et al., 2025). Therefore, AI literacy could be an important moderating variable affecting the effectiveness of AI assistants.

Drawing on SDT (Deci & Ryan, 2000; Ryan & Deci, 2020), this study develops a theoretical framework to explain how perceived AI assistant intelligence may influence medical students’ mental health. In particular, the present study explores the association between perceived AI assistant intelligence and study goal progress, tests the serial mediating roles of study goal progress and academic anxiety on the link between perceived AI assistant intelligence and mental health, and then examines the moderating role of AI literacy in the relationship between perceived AI assistant intelligence and study goal progress. The theoretical model of this study is shown in Figure 1. This study enriches the theoretical framework of research on the application of AI in education and mental health. It offers medical schools guidelines for better optimizing the AI learning support environment, improving students’ AI literacy, and supporting mental health development.

## 2. Theoretical Framework and Hypotheses

### 2.1. Perceived AI Assistant Intelligence and Study Goal Progress

Perceived AI assistant intelligence refers to a user’s overall appraisal of an AI assistant’s apparent capacity to understand instructions, process information, provide accurate and contextually appropriate responses, and adapt its interactions to support the user’s tasks (Bartneck et al., 2009; Ling et al., 2025). The construct therefore concerns capabilities that users attribute to the AI system rather than the user’s evaluation of his or her own learning outcomes. By contrast, study goal progress refers to the user’s perceived advancement toward personally relevant academic goals. Although both constructs were assessed through self-report in Study 1, they differ in their referent and substantive content. Study goal progress is an individual’s perception of how much he or she is progressing toward an ideal academic goal, based on his or her performance and expected results (Klug & Maier, 2015). For medical students, study goal progress reflects how well a student is progressing toward expected learning goals (based on the medical curriculum/medical competency objectives) in knowledge mastery, clinical reasoning, skills training, case analysis, self-directed learning, and periodic assessments (Frank et al., 2010; Holmboe et al., 2010). Previous studies show that the main benefits of AI in medical education include personalized learning support, instant feedback, virtual patient simulations, clinical reasoning training, learning resource creation, and performance analysis (Abd-Alrazaq et al., 2023; Gordon et al., 2024). These features can help medical students identify knowledge gaps early on, adjust their learning strategies, and access learning pathways that better match their needs (Meng et al., 2024; Preiksaitis & Rose, 2023). Therefore, more intelligent AI assistants are more likely to deliver timely, accurate, and actionable learning support that helps medical students feel more purposeful and more on track towards their learning objectives when learning complex medical knowledge and interacting with clinical scenarios.

SDT explains sustained engagement in learning as fulfilling three basic psychological needs: autonomy, competence, and relatedness (Deci & Ryan, 2000; Ryan & Deci, 2020). From an SDT perspective, intelligent AI assistants may support study progress by meeting students’ basic psychological needs. On the other hand, such AI assistants could boost medical students’ independence by providing personalized recommendations and alternative learning paths, helping students feel in control of the learning process (Baidoo-Anu & Ansah, 2023). Moreover, highly intelligent AI assistants may enhance medical students’ sense of competence by providing accurate, timely feedback, step-by-step guidance, and corrective feedback, making it easier for them to feel competent (Kasneci et al., 2023). Moreover, more intelligent AI assistants may foster relatedness and support in learning environments through natural language interaction, responsive feedback, and continuous learning support (Howard et al., 2021). These psychological needs may further foster autonomous motivation, learning engagement, and self-regulation, which are closely associated with academic performance and positive learning outcomes (Bureau et al., 2022). As a result, the more intelligent the AI assistant is, the greater its potential to improve medical students’ learning engagement and self-regulation by satisfying their needs for autonomy, competence, and relatedness, thereby improving learning goal achievement. From the above analysis, the following hypothesis is put forward for this study:

**H1.**  *Perceived AI assistant intelligence is positively associated with study goal progress.*

### 2.2. The Chain Mediation Effect of Study Goal Progress and Academic Anxiety

Academic anxiety is the anxiety, tension, and feeling of helplessness that students experience when coursework, exams, or competency assessments confront them. It usually results from an individual’s evaluation of what is required in the academic field, the chances of failure, and their own coping skills (Pekrun, 2006). According to the World Health Organization (WHO), mental health is a state of physical, mental, and social well-being and not merely the absence of mental illness but also the existence of psychological functioning and subjective well-being (Topp et al., 2015). Medical students are constantly exposed to a high-stress, high-competition, high-responsibility learning environment, which is shown to increase their risk of anxiety, depression, stress, and burnout (Almutairi et al., 2022; Avila-Carrasco et al., 2023; Dyrbye & Shanafelt, 2016; Haykal et al., 2022; Quek et al., 2019).

AI assistants can give feedback, individual explanations, and suggestions for learning courses promptly. This helps medical students recognize knowledge gaps more quickly and use that information to break complex tasks into transparent steps (Gordon et al., 2024; Preiksaitis & Rose, 2023). Students’ sense of competence grows, and uncertainty in learning diminishes as students get closer to the course objectives or competency goals. SDT assumes that satisfaction of a sense of competence, autonomy, and relatedness is essential for positive motivation and psychological well-being (Deci & Ryan, 2000; Ryan & Deci, 2020). This sense of control and efficacy that comes from achieving goals also promotes well-being and continued involvement (Sheldon & Elliot, 1999).

Moreover, this goal achievement in the study may minimize academic anxiety in medical students. As students’ autonomy and competence grow, they are more likely to perceive course stress as a manageable challenge rather than a threat (Almutairi et al., 2022). Conversely, when students do not make progress toward their study goals over time, they are likely to develop negative expectations of failure, recurring concerns, and avoidance behaviors. Previous studies have found that psychological distress, anxiety, stress, and burnout can negatively impact medical students’ academic involvement and achievement, as well as their risk of psychological distress (Dyrbye & Shanafelt, 2016; Rotenstein et al., 2016). Academic stress is closely linked to negative emotional states in the medical learning environment, such as anxiety and depression (Avila-Carrasco et al., 2023; Jia et al., 2022; Quek et al., 2019). Thus, AI assistants perceived as highly intelligent may not only improve students’ immediate learning experiences but also enhance their sense of competence and control by helping them make progress toward their study goals. Therefore, the more positive students’ progress toward study goals, the less worried and concerned they are about studying; and the less worried and concerned they are about studying, the better their mental health. The above analysis has led this study to suggest the following hypothesis:

**H2.**  *Study goal progress and academic anxiety serially mediate the relationship between perceived AI assistant intelligence and mental health.*

### 2.3. The Moderating Role of AI Literacy

AI literacy encompasses understanding the fundamental concepts, capabilities, and limitations of AI; effectively using AI tools; assessing the quality of AI-generated content; and engaging in reflective use with awareness of ethical and safe AI use (Lintner, 2024; Long & Magerko, 2020; X. Wang et al., 2023). In medical education, AI literacy also involves medical students’ capacity to understand AI’s limitations in medical learning and clinical decision-making, and their ability to evaluate information from AI tools rather than taking it on faith or following it mechanically (Laupichler et al., 2024; Pupic et al., 2023).

The SDT states that the external environment is not necessarily conducive to individual development (Deci & Ryan, 2000; Ryan & Deci, 2020). Only when external resources are seen as helping to satisfy the three universal human needs—autonomy, competence, and relatedness—are positive motivation and continued engagement more likely to be stimulated. Thus, the effect of AI assistant support on progress toward study goals depends on whether students can make the support understandable, controllable, and internalizable (Lintner, 2024). AI literacy has a pivotal role in this transformation process.

Therefore, this study suggests that increased AI literacy among medical students would help them better understand AI’s limitations and manage its use more actively. In these situations, students are more likely to view highly intelligent AI assistants as learning aids that support self-regulated, self-directed learning rather than as superior entities that take over their reasoning (Bureau et al., 2022). This enables students to choose how to use AI to achieve their desired learning outcomes, thereby enhancing their sense of autonomy. Meanwhile, students with high AI literacy can better ask appropriate questions, evaluate the quality of AI-generated responses, and incorporate feedback. This allows for the support offered by AI to be more readily translated into knowledge, understanding, and problem-solving skills, and thus increases their sense of competence (Howard et al., 2021). Additionally, natural language interaction, continuous feedback, and companion-like support are more likely to be experienced as supportive relationships, at least to some degree, in the learning process, thereby fulfilling a sense of belonging to some extent (Kong et al., 2025). These three basic psychological needs, in turn, contribute to autonomous motivation, engagement in learning, and self-regulation, which lead to progress toward study goals.

On the other hand, low AI literacy among medical students could hinder the realization of the educational potential of highly intelligent AI assistants. Students might struggle to formulate precise questions and assess the reliability of AI answers (Laupichler et al., 2024). AI feedback can, in these instances, not only fail to promote feelings of competence but also exacerbate confusion and cognitive load. Students may also accept answers without understanding how AI works, which affects their autonomy. Studies show that medical students’ AI literacy is strongly linked to their attitudes toward AI, confidence in AI use and risk perception, and appropriate use of AI tools (Schepman & Rodway, 2023). Thus, higher AI literacy may enable medical students to use intelligent AI assistants more effectively, allowing AI-based support to translate more readily into progress toward study goals. This study proposes the following hypothesis based on this:

**H3.**  *AI literacy moderates the relationship between perceived AI assistant intelligence and study goal progress. When AI literacy is high, the positive correlation between perceived AI assistant intelligence and study goal progress is stronger.*

### 2.4. Moderated Chain Mediation Effect

According to SDT (Deci & Ryan, 2000; Ryan & Deci, 2020), not only the external resources themselves but also the extent to which a person’s basic psychological needs are met influence mental health. With support for autonomy, competence, and relatedness, individuals tend to develop autonomous motivation, positive emotions, and good psychological adaptation, whereas unmet needs foster stress, anxiety, and psychological distress (Howard et al., 2021; Ryan & Deci, 2020). Thus, AI literacy may moderate the relationship between perceived AI assistant intelligence and study goal progress and, consequently, the indirect relationship between perceived AI assistant intelligence and mental health through study goal progress and academic anxiety.

Highly intelligent AI assistants are more likely to be a need-supportive resource for medical students who possess high AI literacy. By having the option to use AI, students can feel in control of the learning process and meet their need for autonomy (Kong et al., 2025). Furthermore, they can more easily leverage AI feedback to identify learning gaps, deepen their knowledge, and adjust learning strategies, thereby improving their competence (Testa et al., 2026). As students make progress toward their learning goals, they gain a clearer sense of advancement toward course objectives and competency standards. This sense of achievement further bolsters their sense of ability, thereby decreasing anticipation of failure and doubt. Students are less likely to be extremely anxious when they feel in control of what they are learning to do (Pekrun, 2006). Hence, in an AI-literate environment, AI assistants are more likely to alleviate academic anxiety by helping students move toward their study goals, thereby further enhancing mental health.

On the other hand, when students have little knowledge of AI, they might not be able to convert AI assistance into personal learning materials. The more information highly intelligent AI assistants offer, the more decision pressure and use burden they create. When this happens, students’ autonomy and sense of competence may not be realized, and the progress toward students’ study goals may be limited (Almutairi et al., 2022). Students’ academic anxiety can still be high if they do not know whether they have mastered the knowledge or not (Haykal et al., 2022). Previous studies have shown that mental health issues such as anxiety, stress, depression, and burnout among medical students are strongly associated with their academic adaptation and performance and that continued academic stress is a risk factor for mental health issues (Quek et al., 2019). Thus, beyond the direct link between perceived AI assistant intelligence and study goal progress, AI literacy also affects mental health through a chain of indirect links: study goal progress, academic anxiety and mental health. High AI literacy may enable medical students to use AI assistants more effectively when they perceive them as highly intelligent, thereby facilitating study goal progress, reducing academic anxiety, and improving mental health. Thus, the following hypothesis of the present study is proposed:

**H4.**  *AI literacy moderates the indirect effect of perceived AI assistant intelligence on mental health via study goal progress and academic anxiety.*

## 3. Study 1: Questionnaire Survey

### 3.1. Sample and Procedure

This study employed purposive sampling to recruit medical students from universities in multiple regions of China, including Nanjing, Sichuan, and Shanghai. We collected data using a digital questionnaire administered through an online survey platform. Participants accessed and completed the questionnaire electronically. We distributed 1059 questionnaires, and 983 were returned. After excluding invalid questionnaires—such as those not fully completed, failing the attention check questions, or exhibiting obvious homogeneity in responses (e.g., selecting the same option consecutively)—a final total of 721 valid questionnaires was obtained.

At the beginning of the questionnaire, the researchers provided all participants with a detailed explanation of the study’s objectives, the scope of data usage, and confidentiality commitments. Participants were explicitly informed that participation was entirely voluntary and that they could opt out of the survey at any stage. To ensure sample validity, we included a screening question before the main questionnaire: “Do you use AI tools in your daily studies?” Respondents who selected “No” were automatically excluded.

Of the 721 respondents, 255 were male (35.4%). Among them, 12.1% were first-year undergraduates, 17.2% were second-year undergraduates, 19.1% were third-year undergraduates, 13.3% were fourth-year undergraduates, and 38.3% were graduate students. The average age was 22.33 years.

### 3.2. Measurement

The measurement tools used in this study were selected based on the following criteria: First, they were sourced from internationally authoritative journals and are widely recognized. Second, they have been validated across multiple cultural contexts, including China, and demonstrate good reliability and validity. To enhance the accuracy and cultural appropriateness of the measurement items, this study used the standard “back-translation method” to revise and refine the original scale wording, ultimately developing a 5-point Likert scale questionnaire that captures the core variables. Scores ranging from 1 to 5 indicate the degree of agreement from low to high (1 = “Strongly Disagree,” 5 = “Strongly Agree”). Appendix A provides the specific items.

Perceived AI Assistant Intelligence. In Study 1, perceived AI assistant intelligence was measured using a five-item scale adapted from the perceived-intelligence dimension of the Godspeed Questionnaire (Bartneck et al., 2009). The measure captured participants’ evaluations of the capabilities displayed by the AI assistant they regularly used; it did not assess participants’ learning performance or goal attainment. A sample item is “The AI learning assistant I currently use has a high level of intelligence.” All items were rated on a five-point Likert scale, with higher scores indicating greater perceived AI assistant intelligence. Cronbach’s alpha was 0.839.

AI literacy. AI literacy is measured using a unidimensional scale with 6 items; higher scores indicate higher AI literacy (Ng et al., 2021). Building on existing research on AI literacy, this study adapted and modified the scale for the medical education context, primarily referencing the AI literacy framework (Ng et al., 2021). Sample items include “I can determine whether AI-generated content is reliable.” The internal consistency coefficient of this scale in this study was 0.867.

Study Goal Progress. Study goal progress is measured using a unidimensional scale with three items; higher scores indicate greater study goal progress (Wanberg et al., 2010). Sample items include “I have made good progress toward completing my study goals.” In this study, the scale’s internal consistency coefficient was 0.880.

Academic Anxiety. Academic anxiety was measured using a 7-item scale; higher scores indicate higher levels of academic anxiety (Liu & Lu, 2012). Sample items include “I consider homework to be a burden.” In this study, the scale’s internal consistency coefficient was 0.853.

Mental health. Mental health was measured using the Patient Health Questionnaire-8 (PHQ-8 (Kroenke et al., 2009)) to assess individuals’ depression-related mental health status and levels of emotional distress. This study utilized the 8-item version of the scale, which assesses symptoms such as loss of interest, depressed mood, sleep problems, fatigue, changes in appetite, diminished self-esteem, difficulty concentrating, and psychomotor retardation or agitation. To ensure consistent scoring direction, all items were reverse-scored in this study, such that higher scores indicate better mental health (i.e., lower levels of psychological distress). The internal consistency coefficient of this scale in this study was 0.951.

Control Variables. Drawing on existing research on individual study goal progress, academic anxiety, and mental health (Adachi et al., 2025; Hao et al., 2026; He et al., 2026), this study included gender, age, and grade level as control variables to prevent them from interfering with the study’s conclusions.

### 3.3. Data Analysis

#### 3.3.1. Common Method Bias Test and Confirmatory Factor Analysis

Although participants completed the questionnaires via self-report, this study tested for common-method bias to strengthen the conclusions (Podsakoff et al., 2003). First, the study used Hamman’s one-factor method to test for common-method bias. The results showed that the first unrotated factor explained 31.562% of the total variance, which was below 50%. Additionally, this study utilized AMOS 24.0 to test for common-method factors, treating them as latent variables within the structural equation model. The findings revealed that the model fit indices did not improve significantly (ΔCFI = 0.003, ΔTLI = 0.004, ΔRMSEA = 0.004, ΔSRMR = 0.006). This indicates that common method bias in this study is not severe.

This study employed AMOS 24.0 to conduct confirmatory factor analysis, as shown in Table 1. The fit indices of the five-factor model (*χ*^2^/df = 2.924, CFI = 0.954, TLI = 0.946, RMSEA = 0.052, SRMR = 0.059) were significantly better than those of other competing models, indicating that the variables possess good discriminant validity and belong to distinct constructs.

#### 3.3.2. Hypothesis Testing

Descriptive statistics were conducted on the means and standard deviations of each variable. Additionally, Table 2 presents the correlation analysis of the variables. This provides preliminary support for our hypotheses. We then used the SPSS PROCESS 4.1 procedure to test the hypotheses.

Main effect test: As shown in Figure 2, there is a significant positive relationship between perceived AI assistant intelligence and study goal progress (B = 0.512, SE = 0.043, *p* < 0.001), supporting H1.

Mediation effect test: This study employed the bootstrap method to test for mediation and moderation effects. With 5000 bootstrap repetitions, the results are shown in Table 3. The indirect effect of perceived AI assistant intelligence on mental health via study goal progress and academic anxiety was significant (B = 0.098, SE = 0.015), with a 95% confidence interval of [0.071, 0.129] that excludes zero, indicating that the mediating effect holds. H2 is supported.

Moderation effect test: As shown in Figure 2, the interaction term between perceived AI assistant intelligence and AI literacy significantly influences study goal progress (B = 0.155, SE = 0.046, *p* < 0.01), supporting H3. Next, this study conducted a simple slopes analysis, with the results shown in Figure 3. Figure 3 indicates that AI literacy strengthens the positive relationship between perceived AI assistant intelligence and study goal progress. Compared with low AI literacy, the positive relationship between perceived AI assistant intelligence and study goal progress is stronger at high AI literacy.

Moderated mediation effect Test: This study employed the bootstrap method to conduct 5000 bootstrap samples to test for a moderated mediation effect. As shown in Table 3, when AI literacy is high, the indirect effect of perceived AI assistant intelligence on mental health via study goal progress and academic anxiety is 0.124, with a 95% confidence interval of [0.090, 0.163], which does not include zero. This indicates that when AI literacy is high, perceived AI assistant intelligence significantly influences mental health via study goal progress and academic anxiety. When AI literacy is low, the indirect effect of perceived AI assistant intelligence on mental health via study goal progress and academic anxiety is 0.072 (95% CI: [0.045, 0.102]), which does not include 0. This indicates that when AI literacy is low, perceived AI assistant intelligence significantly influences mental health through study goal progress and academic anxiety. Furthermore, the difference between the high- and low-score groups is significant (B = 0.052; 95% confidence interval: [0.023, 0.087]). This indicates that the moderated mediating effect holds, thereby supporting H4.

## 4. Study 2: Experimental Study

To enhance situational authenticity and external validity, and to provide evidence beyond the cross-sectional self-report design of Study 1, this study conducted a behavioral experiment in a Chinese context to re-examine the proposed relationships through a real human-AI interaction task.

### 4.1. Experimental Sample and Procedure

This study recruited medical students interested in the research via social media and collected 398 valid questionnaires. All participants had prior experience with AI, as verified by screening questions. Before the experiment began, participants first completed an electronic informed consent form; those who selected “no” were excluded. Regarding sample composition, males accounted for 38.7% and females for 61.3%. In subsequent analyses, this study continued to treat gender, age, and academic year as control variables to mitigate potential confounding effects.

The experimental task was a medical learning–oriented paragraph-revision task. Participants were presented with a standardized short passage describing common basic medical or clinical knowledge, such as the etiology, typical symptoms, diagnosis, and basic treatment of hypertension or type 2 diabetes. They were instructed to use the assigned AI assistant to reorganize the passage, improve its logical structure and clarity, and expand the content where necessary while preserving the core medical information. Participants could interact with the AI assistant during the task to request explanations, revisions, or suggestions. The task simulated a typical AI-assisted learning activity in which medical students use generative AI to process, organize, and refine medical learning materials.

Study 2 manipulated the AI assistant capability condition as an experimental antecedent intended to induce different levels of perceived AI assistant intelligence. Participants were randomly assigned to either a higher-capability model condition (GPT-4) or a lower-capability model condition (GPT-3.5). The GPT versions served as the operational manipulation of assistant capability; they were not treated as the conceptual definition of perceived AI assistant intelligence. After completing the learning task, participants rated the assigned AI assistant’s perceived intelligence using the same Godspeed-based measure employed in Study 1. These ratings were used to evaluate whether the capability manipulation induced different levels of perceived AI assistant intelligence, and the randomly assigned model condition served as the independent variable in subsequent hypothesis tests. The experimental procedure consisted of three stages. First, participants were randomly assigned to either the GPT-4 or GPT-3.5 condition and completed the medical paragraph-revision task using the assigned AI assistant. Second, after completing the task, participants completed the perceived AI assistant intelligence measure as a manipulation check, followed by measures of study goal progress, academic anxiety, and mental health. Finally, participants provided demographic information, including gender, age, and grade level. To align outcome measures temporally with the experimental manipulation, Study 2 administered them immediately after participants completed the AI-assisted learning task and framed them in reference to their current, task-proximal experience. Specifically, study goal progress captured participants’ perceived progress toward the learning goals of the just-completed task, academic anxiety reflected their immediate anxiety and pressure experienced during or immediately after the task, and the mental-health items were framed as post-task assessments of participants’ current psychological state rather than their longer-term mental-health status. Accordingly, the Study 2 measures should be interpreted as short-term, task-proximal indicators following the experimental intervention, rather than as assessments of enduring academic or clinical states. Appendix B provides the full wording of the Study 2 measures.

### 4.2. Manipulation Check

The manipulation check assessed whether the AI capability condition induced the intended difference in participants’ perceived AI assistant intelligence. After completing the task, participants completed the same five-item Godspeed-based perceived-intelligence measure used in Study 1. An independent-samples t-test showed that participants in the higher-capability AI condition reported significantly higher perceived AI assistant intelligence (M = 3.88, SD = 1.057) than those in the lower-capability AI condition (M = 2.79, SD = 1.134), t(396) = 9.879, *p* < 0.001. These results indicate that the capability manipulation successfully generated the intended difference in perceived AI assistant intelligence.

### 4.3. Experimental Results

#### 4.3.1. Reliability and Validity Analysis

First, this study conducted a reliability analysis of the scales using SPSS 27.0. The internal consistency coefficients for AI literacy, study goal progress, academic anxiety, and mental health were 0.876, 0.885, 0.838, and 0.952, respectively, all exceeding 0.7, indicating good reliability. This study then used AMOS 24.0 to conduct confirmatory factor analysis and assess discriminant validity among variables. Compared with competing models, the four-factor model showed the best fit (*χ*^2^/df = 1.630, *p* < 0.001; CFI = 0.978; TLI = 0.975; RMSEA = 0.040; SRMR = 0.061), indicating that the research model has good discriminant validity.

#### 4.3.2. Hypothesis Testing

The following analyses therefore concern post-task, task-proximal outcomes assessed immediately after the experimental manipulation.

First, ANOVA compared study goal progress, academic anxiety, and post-task mental-health scores between the higher- and lower-capability AI conditions, which the manipulation check showed induced higher versus lower perceived AI assistant intelligence. Figure 4 shows the results. Study goal progress was significantly higher in the higher-capability AI condition (M = 3.30, SD = 1.166) than in the lower-capability AI condition (M = 2.54, SD = 1.022; F(1, 396) = 47.249, *p* < 0.001, ηp^2^ = 0.107). Academic anxiety was significantly lower in the higher-capability AI condition (M = 2.54, SD = 0.868) than in the lower-capability AI condition (M = 3.12, SD = 0.817; F(1, 396) = 46.410, *p* < 0.001, ηp^2^ = 0.105). Post-task mental health was significantly higher in the higher-capability AI condition (M = 3.51, SD = 1.087) than in the lower-capability AI condition (M = 2.68, SD = 0.987; F(1, 396) = 62.864, *p* < 0.001, ηp^2^ = 0.137).

To test the hypotheses in Study 2, we dummy-coded the experimentally assigned AI capability condition (higher-capability condition = 1, lower-capability condition = 0) and entered it into the analyses using SPSS PROCESS 4.1.

Main effect test: As shown in Figure 5, the experimentally assigned AI capability condition significantly predicted study goal progress (B = 0.847, SE = 0.110, *p* < 0.001). Because the manipulation check showed that this condition successfully induced different levels of perceived AI assistant intelligence, this result provides experimental support for H1.

Mediation effect test: This study employed the bootstrap method to test the indirect-effect model. Table 4 shows the results based on 5000 bootstrap samples. The indirect effect of the experimentally assigned AI capability condition on post-task mental health through study goal progress and academic anxiety was significant (B = 0.108, SE = 0.032), with a 95% confidence interval of [0.048, 0.173] that did not include 0. Given that the manipulation check confirmed differences in perceived AI assistant intelligence between conditions, this finding is consistent with the proposed serial mediation mechanism and supports H2.

Moderation effect: As shown in Figure 5, the interaction term between the experimentally assigned AI capability condition and AI literacy significantly predicted study goal progress (B = 0.368, SE = 0.060, *p* < 0.01), supporting Hypothesis 3. Next, this study conducted a simple slopes analysis, with the results shown in Figure 6. Figure 6 indicates that AI literacy strengthened the positive effect of the higher-capability AI condition on study goal progress. Compared with participants with low AI literacy, participants with high AI literacy benefited more from the higher-capability AI condition in terms of study goal progress.

Moderated mediation effect: This study employed 5000 bootstrap samples to test the moderated mediation model. As shown in Table 4, when AI literacy was high, the indirect effect of the experimentally assigned AI capability condition on post-task mental health through study goal progress and academic anxiety was 0.152 (95% CI: [0.066, 0.242]), which excluded zero. When AI literacy was low, the corresponding indirect effect was 0.065 (95% CI: [0.017, 0.125]), which also excluded zero. Furthermore, the difference between the conditional indirect effects was significant (B = 0.086; 95% confidence interval: [0.021, 0.168]). Given that the capability manipulation induced different levels of perceived AI assistant intelligence, these results are consistent with the proposed moderated serial mediation model and support H4.

## 5. Discussion

### 5.1. Theoretical Significance

First, this study extends research on learners’ psychological outcomes in AI applications in education. Existing studies have mainly focused on using AI to improve learning efficiency, academic outcomes, and technology use, highlighting AI’s functional value as a learning tool (Kasneci et al., 2023; Preiksaitis & Rose, 2023; Venkatesh et al., 2003). In contrast, research on the impact of AI assistants on learners’ psychological health has been relatively limited. This study uses a medical education context to investigate whether higher perceived AI assistant intelligence influences medical students’ mental health, treating perceived AI assistant intelligence as an important factor in the learning environment. This not only shifts AI education research from a predominant focus on learning performance toward greater attention to mental health but also broadens the spectrum of outcome variables examined in research on AI applications in education (Gordon et al., 2024; Jia et al., 2022; Mohmand et al., 2022).

Second, this study demonstrates the relevance of SDT for understanding AI-mediated learning contexts. Previous SDT studies have mostly examined the effects of conventional educational factors (e.g., teacher support and peer interaction) on motivation to learn and psychological development (Bureau et al., 2022). As AI assistants become more integrated into learning, intelligent systems are becoming a major environmental resource that influences learning (Kasneci et al., 2023). From an SDT perspective, AI assistants perceived as highly intelligent may create a more supportive learning environment by facilitating experiences related to autonomy, competence, and relatedness, which may in turn contribute to study goal progress and psychological adaptation. In this sense, the present findings suggest that SDT provides a useful theoretical lens for understanding how AI-supported learning environments may influence students’ learning and psychological outcomes, thereby extending SDT’s relevance to emerging human–AI collaborative learning settings.

Third, this study clarifies the mechanisms and boundary conditions through which perceived AI assistant intelligence affects medical students’ mental health. While research shows that AI can positively influence learning experiences and outcomes, systematic explanations of how AI affects learners’ mental health remain lacking (Klug & Maier, 2015). This study develops and validates a serial mediation mechanism through which perceived AI assistant intelligence affects mental health, with study goal progress and academic anxiety playing pivotal roles (Pekrun, 2006). In addition, this study includes AI literacy as a moderating variable, indicating that the positive association between perceived AI assistant intelligence and mental health is not universal but depends on individual AI literacy (Ng et al., 2021). This finding contributes to understanding how AI influences the psychological aspects of learning in educational contexts and provides a theoretical basis for further research on individual differences in AI-assisted learning environments.

### 5.2. Practical Implications

First, this study found that higher perceived AI assistant intelligence is associated with improved mental health among medical students by facilitating progress toward study goals and reducing academic anxiety. This suggests that AI in medical education should focus on whether it provides effective learning support (Gordon et al., 2024). Thus, when designing and implementing AI learning aids, medical schools should consider how students perceive the intelligence and learning-support capabilities of AI assistants rather than focusing solely on system usability and adoption, including their ability to understand questions, provide accurate feedback, adapt to different learning situations, and support individual students (Hale et al., 2024). Highly intelligent AI assistants can offer continuous interaction, personalized feedback, and learning guidance, which may be more effective than simple information retrieval or standardized question-and-answer functions in helping medical students progress toward learning goals and maintain a positive psychological state (Meng et al., 2024; Preiksaitis & Rose, 2023).

Second, this study found that study goal progress and academic anxiety play a serial mediating role between perceived AI assistant intelligence and mental health. This indicates that one important way AI assistants perceived as highly intelligent may influence mental health is by supporting students’ progress toward their learning goals (Harkin et al., 2016). Thus, teachers and educational administrators can make greater use of AI assistants for learning planning, milestone management, identifying learning gaps, and providing learning feedback, thereby helping students continuously progress toward their learning goals (Wanberg et al., 2010). For medical students who are continuously exposed to high-pressure learning environments, such support may enhance their sense of control over learning and reduce anxiety arising from academic uncertainty (Pekrun, 2006; Quek et al., 2019).

Third, this study revealed that AI literacy further strengthens the role of perceived AI assistant intelligence in promoting study goal progress. This indicates that even when students interact with AI assistants offering similar levels of support, the extent of their benefit may differ depending on their AI literacy (Laupichler et al., 2024). Thus, while medical schools improve AI-supported education, they should not only provide the technology but also emphasize developing students’ AI literacy (Long & Magerko, 2020). In particular, AI literacy courses, case-based teaching, and critical evaluation training can help students understand AI’s functional capabilities and limitations, develop effective questioning skills, and critically evaluate AI-generated content (Pupic et al., 2023). Improving students’ AI literacy may therefore help them better translate the learning and psychological benefits of highly intelligent AI assistants into positive learning outcomes.

### 5.3. Limitations and Future Research

While this study has contributed to the literature on the use of artificial intelligence in education and medical students’ mental health, it has weaknesses that future research should address.

First, although this study used a research design integrating experimental techniques and questionnaire surveys, the experimental environment was primarily based on a short-term, simulated AI-assisted learning task, which may not fully reflect medical students’ actual and sustained experiences using AI in educational settings. In addition, Study 2 assessed the outcome variables immediately after the experimental task and framed them as task-proximal, post-intervention states. Therefore, Study 2’s findings should not be interpreted as evidence that a brief interaction with a more capable AI assistant produces enduring changes in students’ study goal progress, academic anxiety, or mental health. In particular, the mental-health measure in Study 2 reflects participants’ immediate psychological state following the task rather than their longer-term or clinical mental-health status. Future studies could use real learning platforms, repeated AI-assisted learning sessions, or longitudinal designs to examine whether these short-term effects persist over time and further enhance the ecological validity of the findings (Gordon et al., 2024). Second, this study mainly collected data through self-report scales, so participants’ subjective perceptions and social desirability may have influenced the variables. Future studies could incorporate more objective indicators, such as academic performance, learning behavior logs, or teacher assessments, to cross-validate findings and strengthen their robustness (Podsakoff et al., 2003). Third, this study examined only study goal progress, academic anxiety, and AI literacy. Future studies could further consider other relevant factors, such as academic self-efficacy, learning engagement, and trust in technology, and test the model across different majors and educational levels to deepen the theoretical explanation of how perceived AI assistant intelligence influences learner development. Finally, this study focused specifically on medical students. Given the high levels of stress, competition, and competency-based training in medical education, it remains unclear whether the findings generalize to other student populations. Future studies could replicate the study across different disciplines, educational levels, and cultural contexts to enhance the external validity and generalizability of the findings.

## Figures and Tables

**Figure 1:**
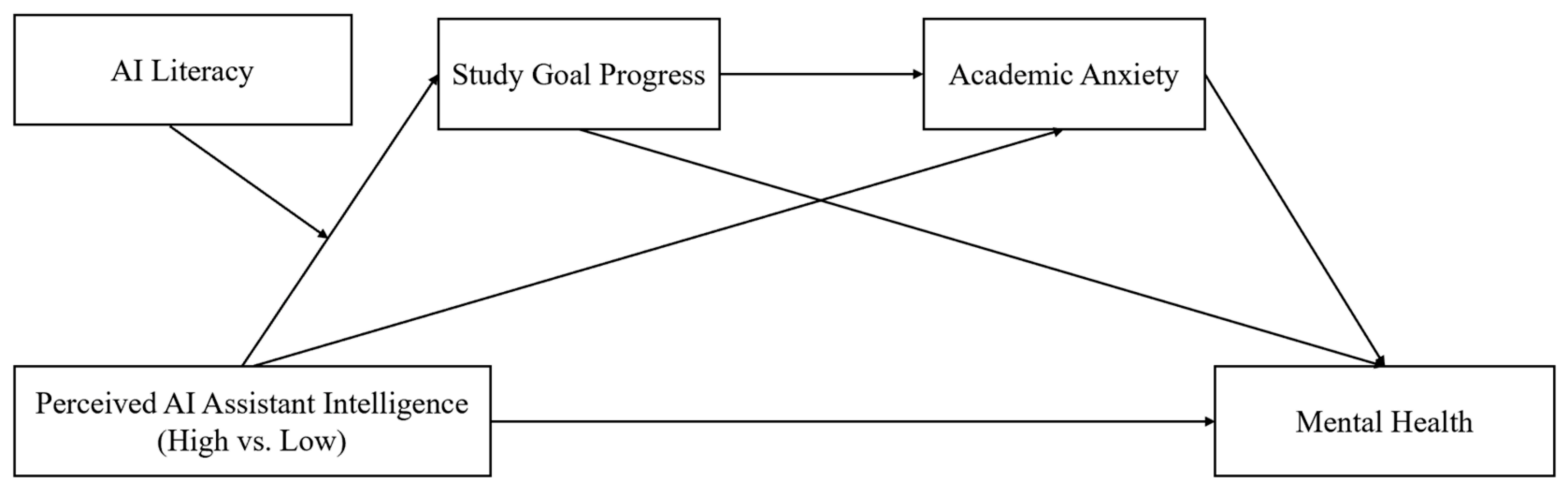
Theoretical model diagram.

**Figure 2:**
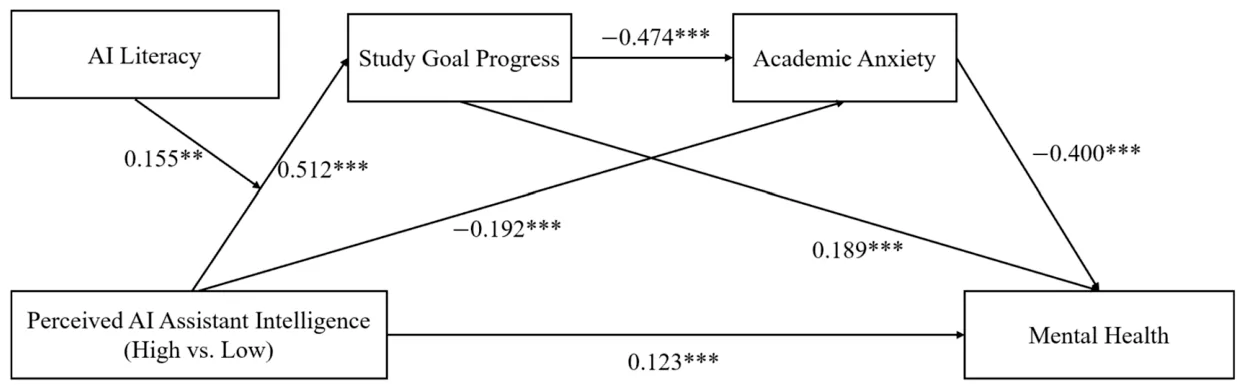
Path Coefficients of the Hypothesized Model in Study 1. Note. B = Path coefficients; SE = Standardized errors; Path coefficients are standardized; ** *p* < 0.01; *** *p* < 0.001.

**Figure 3:**
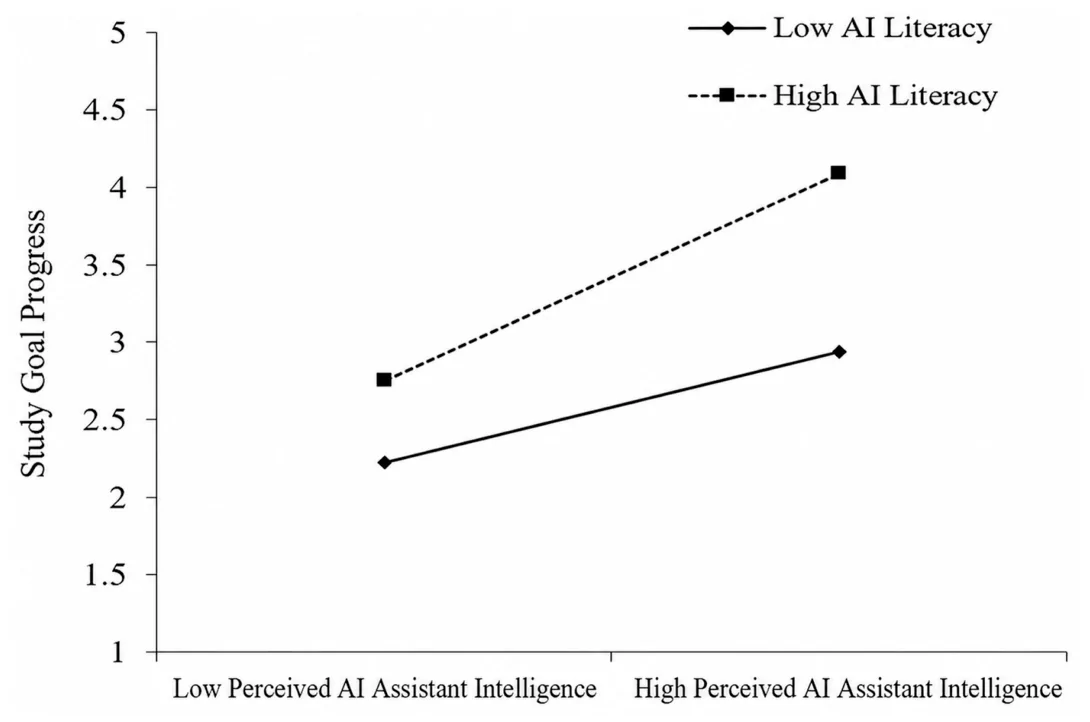
The moderating effect of AI literacy on the relationship between perceived AI assistant intelligence and study goal progress in Study 1.

**Figure 4:**
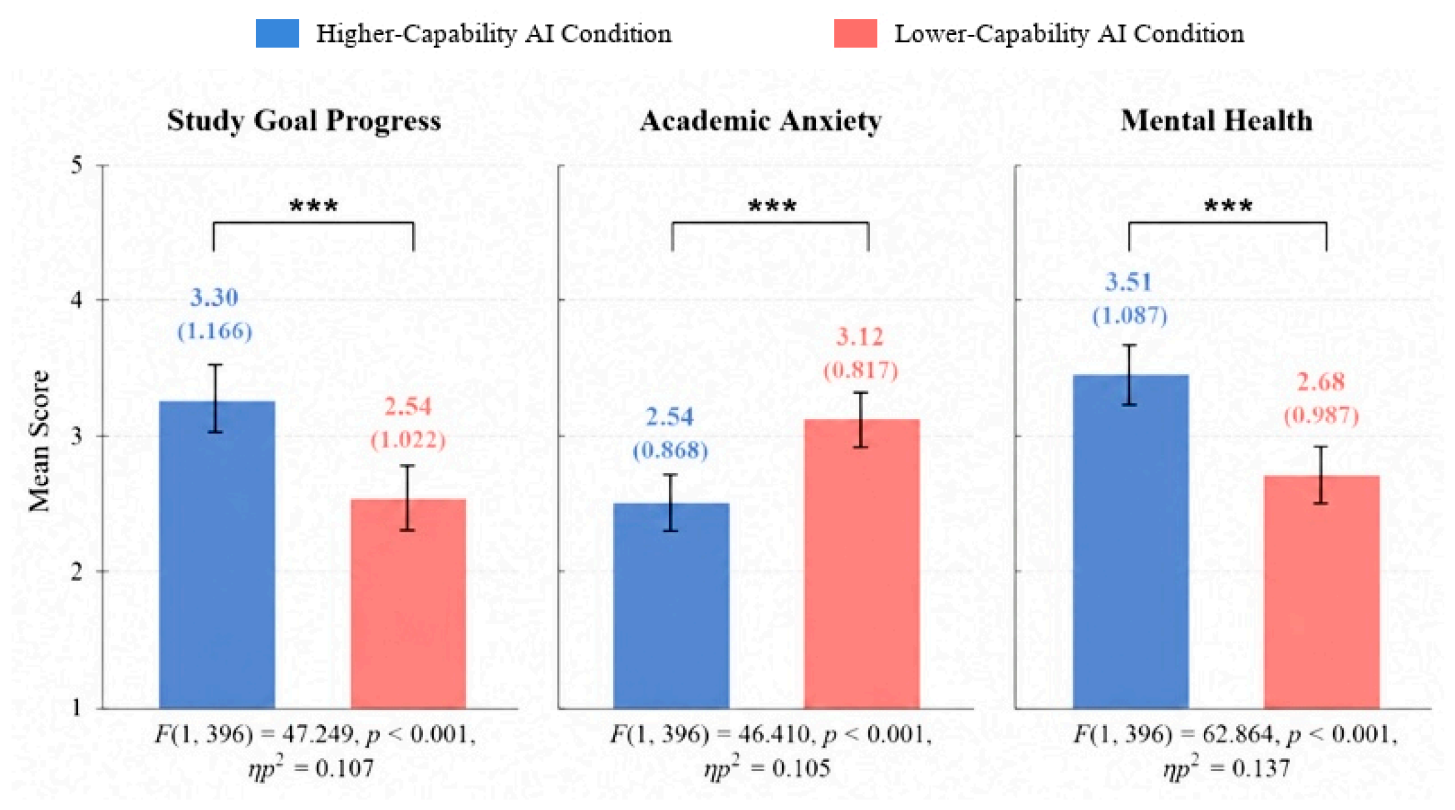
Effects of the experimentally assigned AI capability condition on key outcomes in Study 2. Notes. Values above bars are Mean (SD). Error bars represent standard deviation, *** *p* < 0.001.

**Figure 5:**
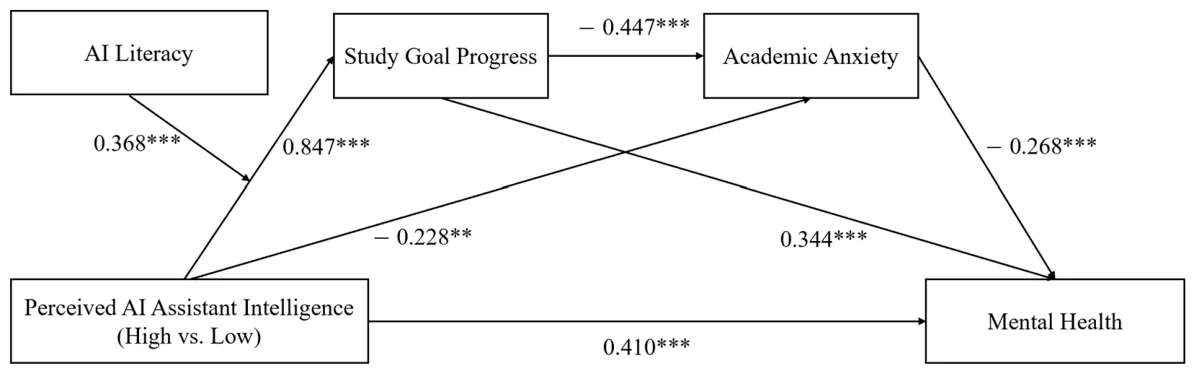
Summary of path-analytic results in Study 2. ** *p* < 0.01; *** *p* < 0.001.

**Figure 6:**
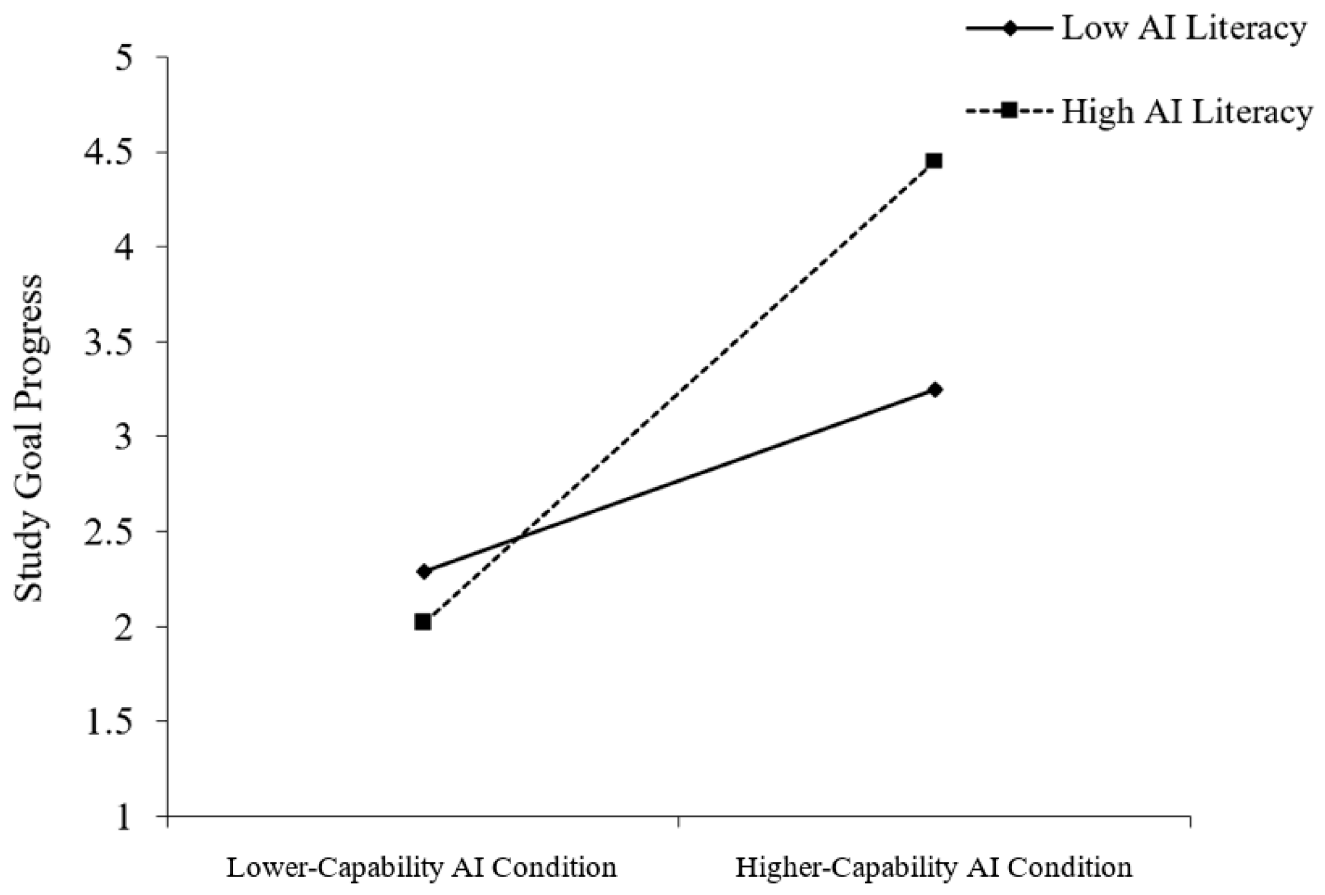
The moderating effect of AI literacy on the relationship between AI capability condition and study goal progress in Study 2.

**Table 1:** Results of confirmatory factor analyses.

Model	Factors	*χ* ^2^	df	*χ*^2^/df	CFI	TLI	RMSEA	SRMR
5-factor model	PAII; AIL; SGP; AA; MH	1005.724	344	2.924	0.954	0.946	0.052	0.059
4-factor model	PAII + AIL; SGP; AA; MH	2497.341	348	7.176	0.851	0.826	0.093	0.121
3-factor model	PAII + AIL + SGP; AA; MH	4128.638	351	11.763	0.738	0.697	0.122	0.156
2-factor model	PAII + AIL + SGP + AA; MH	4022.564	353	11.395	0.746	0.708	0.120	0.147
1-factor model	PAII + AIL + SGP + AA + MH	5924.701	354	16.736	0.614	0.557	0.148	0.167

Note. PAII = Perceived AI Assistant Intelligence; AIL = AI Literacy; SGP = Study Goal Progress; AA = Academic Anxiety; MH = Mental Health.

**Table 2:** Means, standard deviations, and correlations of variables.

Variables	1	2	3	4	5	6	7	8
1. Gender	-							
2. Age	0.120 **	-						
3. Grade	0.061	0.497 **	-					
4. Perceived AI Assistant Intelligence	−0.045	−0.020	−0.025	-				
5. AI Literacy	−0.055	−0.009	0.058	−0.286 **				
6. Study Goal Progress	−0.001	0.012	0.021	0.304 **	0.225 **			
7. Academic Anxiety	0.092 *	−0.006	0.065	−0.378 **	0.013	−0.650 **		
8. Mental Health	−0.071	0.066	−0.014	0.290 **	−0.029	0.445 **	−0.507 **	
Mean	1.65	22.33	3.75	3.2239	2.5546	2.9686	2.8094	3.1810
SD	0.478	2.232	1.415	0.92705	0.87922	1.15066	0.92036	1.10454

Note. N = 721. Gender was coded as 1 = male and 2 = female. Grade level was coded as 1 = freshman, 2 = sophomore, 3 = junior, 4 = senior, and 5 = graduate student. * *p* < 0.05; ** *p* < 0.01.

**Table 3:** Bootstrapping results for testing the mediation effect and moderated mediation effect in Study 1.

Items	B	SE	95% CI Lower	95% CI Upper
Perceived AI Assistant Intelligence → Study Goal Progress → Academic Anxiety → Mental Health				
Indirect effect	0.098	0.015	0.071	0.129
Direct effect	0.120	0.041	0.040	0.200
High (+AIL)	0.124	0.019	0.090	0.163
Low (−AIL)	0.072	0.015	0.045	0.102
Difference	0.052	0.016	0.023	0.087

Note. B = path coefficient; SE = standard error; confidence intervals that exclude zero indicate significant indirect effects; path coefficients are standardized; number of bootstrap samples = 5000; confidence level = 95%.

**Table 4:** Bootstrapping results for testing mediation and moderated mediation effects in Study 2.

Items	B	SE	95% CI Lower	95% CI Upper
AI Capability Condition → Study Goal Progress → Academic Anxiety → Mental Health				
Indirect effect	0.108	0.032	0.048	0.173
Direct effect	0.410	0.096	0.222	0.598
High (+AIL)	0.152	0.044	0.066	0.242
Low (−AIL)	0.065	0.028	0.017	0.125
Difference	0.086	0.038	0.021	0.168

Note. B = path coefficient; SE = standard error; confidence intervals that exclude zero indicate significant indirect effects; path coefficients are standardized; number of bootstrap samples = 5000; confidence level = 95%.

## Data Availability

The data presented in this study are available on request from the corresponding author due to privacy reasons.

## Appendix A. Items in Study 1

Perceived AI Assistant Intelligence (Bartneck et al., 2009)

1. The AI learning assistant I currently use is highly capable.
2. The AI learning assistant I currently use has extensive knowledge.
3. The AI learning assistant I currently use performs reliably in learning tasks.
4. The AI learning assistant I currently use has a high level of intelligence.
5. The AI learning assistant I currently use is capable of making sound judgments.

AI Literacy (Ng et al., 2021)

1. I understand the basic principles underlying how generative AI tools work.
2. I can effectively use AI tools to support my medical learning.
3. I can determine whether AI-generated content is reliable.
4. I understand the limitations of AI tools in medical learning.
5. I can use AI tools appropriately according to my learning needs.
6. I understand the ethical and academic standards that should be observed when using AI.

Study Goal Progress (Wanberg et al., 2010)

1. I have made good progress toward achieving my learning goals.
2. I am highly efficient and productive in completing my learning tasks.
3. I continuously work toward and accomplish my established learning goals.

Academic Anxiety (Liu & Lu, 2012)

1. Completing assignments makes me feel stressed.
2. I consider assignments to be a burden.
3. I have a large number of assignments to complete.
4. Classroom learning tasks are difficult for me.
5. I often have to learn material in class that is difficult to understand.
6. Examinations are generally very difficult for me.
7. I find it difficult to complete the problem-solving tasks assigned by my teachers.

Mental Health (Kroenke et al., 2009)

1. I have little interest or pleasure in engaging in daily activities.
2. I feel down, depressed, or hopeless.
3. I have difficulty falling asleep, staying asleep, or sleep too much.
4. I feel tired or have little energy.
5. I experience poor appetite or overeating.
6. I feel dissatisfied with myself or believe that I am a failure.
7. I have difficulty concentrating.
8. I move or speak unusually slowly, or I become noticeably restless or agitated.

## Appendix B. Items in Study 2

AI Literacy (Ng et al., 2021)

1. I understand the basic principles underlying how generative AI tools work.
2. I can effectively use AI tools to support my medical learning.
3. I can determine whether AI-generated content is reliable.
4. I understand the limitations of AI tools in medical learning.
5. I can use AI tools appropriately according to my learning needs.
6. I understand the ethical and academic standards that should be observed when using AI.

Study Goal Progress (adapted from Wanberg et al., 2010)

1. After completing this AI-assisted learning task, I believe that I made good progress toward the learning goal of the task.
2. During the preceding learning task, I was able to work efficiently toward and complete the intended learning content.
3. Overall, I believe that I made good progress toward the predefined goals of this learning task.

Academic Anxiety (adapted from Liu & Lu, 2012)

1. Completing the preceding medical learning task made me feel stressed.
2. I felt nervous while completing the preceding learning task.
3. The preceding learning task placed a degree of psychological burden on me.
4. While completing the preceding learning task, I was concerned that I might not meet the task requirements successfully.
5. I found some of the material in the preceding learning task difficult to understand.
6. While completing this task, I felt concerned about my learning performance.
7. Overall, completing the preceding learning task made me feel anxious.

Mental Health (Post-task Psychological State; adapted from Kroenke et al., 2009)

1. After completing this AI-assisted learning task, I remain interested in the learning activities that follow.
2. After completing this task, I currently feel positive and emotionally stable.
3. After completing this task, I currently feel relatively relaxed rather than tense or restless.
4. After completing this task, I still feel that I have enough energy to continue learning.
5. After completing this task, I currently do not feel noticeably discouraged or helpless.
6. After completing this task, I generally feel positive about my learning performance.
7. After completing this task, I am currently able to concentrate well.
8. Overall, I am currently in a good psychological state after completing this task.
